# Agreement between seroprevalence- and model-based estimates of COVID-19 burden

**DOI:** 10.1080/16549716.2026.2683286

**Published:** 2026-06-09

**Authors:** Nana Owusu-Boaitey, Lucas Böttcher, Gideon Meyerowitz-Katz, Jonathan Howard, David H. Gorski, Lonni Besançon, Anton Barchuk

**Affiliations:** a Fischell Department of Bioengineering, University of Maryland, College Park, MD, USA; b Department of Computational Science and Philosophy, Frankfurt School of Finance and Management, Frankfurt, Germany; c Department of Medicine, University of Florida, Gainesville, FL, USA; d School of Nursing, Faculty of Science, Medicine and Health, University of Wollongong, Wollongong, Australia; e Departments of Psychiatry and Neurology, New York University Langone School of Medicine, New York, NY, USA; f Departments of Surgery and Oncology, Wayne State University School of Medicine, Detroit, MI, USA; g Media and Information Technology, Linköping University, Norrköping, Sweden; h European University at St. Petersburg, St. Petersburg, Russia; i ITMO University, St. Petersburg, Russia

**Keywords:** COVID-19, seroprevalence, infection fatality rate, epidemiological modeling, mortality burden

## Abstract

The severe acute respiratory syndrome coronavirus 2 (SARS-CoV-2) pandemic caused significant global harm. Seroprevalence studies detected antibody increases from SARS-CoV-2 infection, assessing transmission and risk from infection. These studies and epidemiological modeling informed public health policy and behavior. Biases in early seroprevalence analyses caused underestimation of fatality risk, including the risk posed to pediatric populations and to those in lower-income nations. The scope of the pandemic was clarified by seroprevalence studies that better assessed at-risk groups and by adjustment of seroprevalence data for known data biases. Reassessment of seroprevalence data confirms the accuracy of early modeling. Public health authorities should inform the public of this modeling success and of the limitations of early seroprevalence analyses. This will prepare the public to better assess global health risk during future pandemics.

## Paper context

- **Main findings**: Limitations of early seroprevalence analyses contributed to underestimation of risk.
- **Added knowledge**: Early modeling accurately represented SARS-CoV-2 transmission and fatality risk.
- **Global health impact for policy and action**: Health authorities should inform the public of success in modeling and limitations of seroprevalence analyses early in future outbreaks.

## Background

Serology revealed the global health burden of the severe acute respiratory syndrome coronavirus 2, SARS-CoV-2, pandemic. Serological testing estimated seroprevalence, the prevalence of SARS-CoV-2-specific antibodies. Seroprevalence was then used to infer cumulative incidence of infection, fatality rate from infection, and hospitalization rate from infection (CI, IFR, and IHR, respectively). These estimates identified demographics at greater risk and informed public policy globally [1,2]. We examine how biases in serological studies shaped understanding of the pandemic and means of mitigating these biases. Epidemiological modeling also provided policy-relevant information before seroprevalence studies were performed [3–7]. We argue that reliable seroprevalence data confirms the accuracy of this modeling and casts doubt on less reliable seroprevalence analyses that underestimated global health risk.

## Sampling bias

Sampling bias reduced the accuracy of SARS-CoV-2 seroprevalence analyses. Seroprevalence studies ideally tested individuals randomly selected from a sample frame representative of the general population. This generated a probability sample when participation rates were sufficiently high [8,9]. Lower participation rates caused results to be less representative. Those who received a SARS-CoV-2-positive test later in the pandemic were less likely to participate in serological studies because they no longer desired confirmation of infection [10]. But early in the pandemic when CI was lower, individuals with more risk factors for infection were more likely to enroll. A desire to check infection and immunity status likely motivated their study participation [6,8,11]. Factors such as distrust in public health researchers may also have contributed to sampling bias [12].

Nonprobability sampling supplied important information early in outbreaks when behavior changes and policy interventions could better limit transmission. Nonprobability sampling used nonrandom selection of participants or sample frames not representative of the general population. Some nonprobability samples approximated the results of random sampling, though this alignment was not guaranteed [6,9]. Nonprobability sampling was most useful when it was longitudinal. This allowed for robust interpretation of CI and ongoing monitoring of key pandemic metrics. The United Kingdom, for example, maintained three nationally representative samples throughout most of the pandemic. These samples proved invaluable for pandemic science [13]. It is unfortunate that more countries were not able to follow suit, especially resource-limited nations [14]. The relative absence of longitudinal seroprevalence assessments obscured the burden and spread of the pandemic in these nations [15,16].

Seroprevalence analyses with nonprobability sampling caused underestimation of the risk posed by SARS-CoV-2. April 2020 seroprevalence studies conducted in Los Angeles and Santa Clara counties in California, for instance, dominated public discourse [12,17,18]. Sood *et al*. implied an IFR of 0.2% in Los Angeles by testing people in a marketing database [7,17]. Bruckner *et al*. also used this database for a seroprevalence study in Orange County, California [19]. Bruckner *et al*. and Sood *et al*. implied lower IFRs and proportion of infections reported than probability sampling elsewhere in California [7,19,20] and through early May 2020 [3,6,7,16]. This suggests individuals selected from the database were not representative of the general population [7,9].

Bendavid *et al*. inferred 0.2% IFR in Santa Clara by testing participants recruited through advertisements [3,18]. Advertisements exacerbated nonresponse bias, skewing CI estimates upward [6,11]. An unknown proportion of the population saw advertisements, precluding determination of response rate and limiting the accuracy of adjustments for nonresponse bias. Nonresponse adjustments were more effective when applied after randomly sampling a well-characterized sample frame with known differences between responders versus nonresponders [8]. Bendavid *et al*. instead adjusted for nonresponse without accounting for important behavioral and demographic differences [9]. Bias from these differences was likely exacerbated by recruiting participants with the suggestion that testing positive mitigated the need for public health restrictions [18].

## Modeling informed the public

Epidemiological modeling provided crucial information in the initial weeks of the pandemic when seroprevalence data was lacking. Modelers inferred IFR from limited data [3,4,21], and public health authorities used these assessments to inform the public. The World Health Organization (WHO) stated a modeled IFR range of 0.3–1% in February 2020, with greater risk for older age groups with underlying health conditions [22,23]. Public health officials used 1% modeled IFR to inform public opinion and project millions of deaths in the absence of measures mitigating transmission [24].

Governments justified lockdown measures using March 2020 modeling from Verity *et al*. and Ferguson *et al*. at Imperial College London [4,21,25]. This modeling projected 0.8% IFR for the USA, with two million deaths across two years in the absence of control measures, vaccines, and behavior changes. Projected IFR, IHR, total deaths, and total hospitalizations ranged between 0.4–0.9%, 2.8–4.3%, 0.6–2.8 million, and 4.4–12.9 million, respectively, under other scenarios [21,25,26]. These projections confirmed SARS-CoV-2 was a greater global health threat than seasonal influenza [6,27]. IFRs of ≤0.2% inferred from nonprobability sampling instead supported projections of ≤40,000 deaths, comparable to influenza [25,28,29].

## Model accuracy

Subsequently published seroprevalence data supported modeled IFR estimates. National and statewide probability samples implied IFR near 1% for the USA [30,31], consistent with the Imperial modeled range of 0.4–0.9%. Modeled IFR of 0.9% for Great Britain was consistent with ~1% IFR inferred from three independent probability samples [6,21,27,32]. Modeled population-wide IFRs were also accurate for lower-income nations such as India, after accounting for underreporting of SARS-CoV-2 deaths [16,26,33–35]. Multinational serological data [3,6,7,15,16,27,32,33,35,36] confirmed the WHO modeled IFR range of 0.3–1% and other modeled estimates published within the first few weeks of the pandemic [3,7,22].

Serological evidence supported modeled projections of global health burden. Serology confirmed greater shielding of the elderly from infection in higher- versus lower-income countries [16,37]. This shielding pattern was predicted by modeling of age-specific contact patterns [4,21,26]. Serology also showed SARS-CoV-2 could achieve the high CI projected in modeled scenarios [14,16,38]. CI and disease burden were limited by factors such as behavior changes, nonpharmaceutical interventions, and vaccination [14]. These factors contributed to 0.5–1% IFR and 3–5% IHR for the USA through 2021, with at least 1 million deaths and 4–7 million hospitalizations [33,36,39]. This mortality and morbidity exceeded influenza by at least an order of magnitude, despite mitigation of the SARS-CoV-2 outbreak [29]. March 2020 modeled projections of two million deaths in an unmitigated outbreak [21,26] therefore provided better guidance than predictions of ≤40,000 deaths based on seroprevalence analyses with nonprobability sampling [25,28].

**Figure 1.**
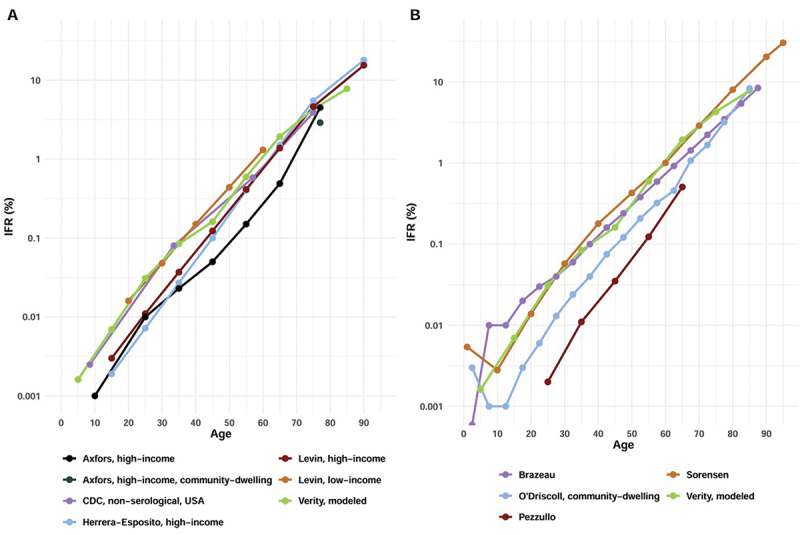
Age-specific infection fatality rates (IFR) from serological and non-serological analyses, *(A)* distinguishing between or *(B)* merging data from high-income and low-income nations. CDC is the only analysis to include individuals vaccinated against SARS-CoV-2. CDC and Verity infer IFR from non-serological data for the USA and China, respectively [4,39]. All other studies are meta-analyses of cross-national seroprevalence results [6,16,27,32,36,38,42,43]. The USA is classified as high-income, and China is classified as low-income [16]. CDC and Sorensen use excess deaths to adjust for underreporting of SARS-CoV-2 deaths. The remaining studies use reported SARS-CoV-2 deaths. ‘Community-dwelling’ studies sample only individuals outside of institutional settings, such as by excluding elderly people in nursing homes. This causes community-dwelling IFR to underestimate population-wide non-age-specific IFR. Pezzullo IFR for age 10 is 0.0003% (not shown). Data is plotted at the midpoint age within each age group and 10 years above the lower bound for the last open age group.

Modeling provided policy-relevant information across age groups. Pediatric infections, hospitalizations, and deaths in the USA exceeded those from influenza [39], consistent with early modeling [4,21,26]. Later serological evidence confirmed IFR increases with age from WHO assessments and the Imperial College modeling of Verity *et al.* (Figure 1) [4,23]. Nonprobability sampling and mortality underreporting likely contributed to underestimated IFR by Axfors *et al*. and Pezzullo *et al.* (Figure 1) [2,16,40]. IFR underestimation was confirmed by pediatric deaths per capita in the USA and Mexico exceeding pediatric IFR from Pezzullo *et al*. before most minors were infected [39,41]. The lower IFR estimates of Pezzullo *et al*. and Axfors *et al*. led to overly pessimistic assessments of the accuracy of modeling performed in Verity *et al* [25,42,43].

## Impact of flawed estimates

Modeling suffered from important limitations. Imperial College modeling, for example, assumed similar age-specific IFR in lower- versus higher-income countries [21,26]. Age-specific IFR was instead greater in lower-income nations [15], as shown by comparing Levin *et al*. and Herrera-Esposito *et al*. in Figure 1A [16,36]. Modeling also incorrectly assumed more transmissible variants would not develop and that postinfection immunity prevented all reinfections [21,44]. Modeled projections could overestimate reported hospitalizations due to underreporting and out-of-hospital deaths [36,39].

Flawed IFR assessments influenced strategies for protecting vulnerable populations. Some commentary proposed shielding the elderly from infection because of higher IFR in this demographic (Figure 1) [25]. Substantial mortality occurred despite some shielding [16,39]. Less shielding in lower-income countries undermined hope that their younger age distribution would sufficiently limit deaths [16,37]. Significant mortality in lower-income nations was also obscured by mortality underreporting and the incorrect assumption of similar IFR in lower- versus higher-income nations (Figure 1A) [15,16]. This mortality could have been mitigated with better understanding of fatality risk and more equitable vaccine access.

IFR analyses impacted vaccination strategies and assessment of vaccination benefits. Underestimated IFR in lower-income nations obscured their urgent need for vaccines. Vaccine doses were instead disproportionately distributed to wealthier nations due to factors such as unequal political power [2]. Nations typically vaccinated older individuals before moving to younger age groups, largely due to increasing IFR with age. Assumed age-specific fatality risk also influenced estimates of deaths averted by vaccination [1,2,45]. IFR estimates from Brazeau *et al*., for instance, implied an order of magnitude more deaths averted than lower IFR estimates from Axfors *et al*. and Pezzullo *et al* (Figure 1) [1,45]. These lower estimates of deaths averted may be less plausible in virtue of nonprobability sampling and mortality underreporting [2,16,40].

## Implications for future outbreaks

Public health communication during future pandemics should emphasize results from reliable seroprevalence analyses. Well-designed probability sampling was performed within the first few months of the pandemic [6,7,16], but garnered less attention than results from less reliable nonprobability sampling [12]. Limitations of early analyses must therefore be clearly communicated when using seroprevalence data to inform the public. As results from probability sampling accumulate, they should replace results from nonprobability sampling, where practical. Early serological testing can include sample frames established before the outbreak and which are representative of the general population [7,13]. Estimates of CI and severity rates should also improve with refinements such as adjustments for antibody waning [27] and nonresponse adjustments encompassing behavioral, socioeconomic, and demographic factors [8]. Without these refinements, early misleading serological analyses may undermine both understanding of the pandemic and planning for future outbreaks.

Outbreak modeling remains crucial for limiting harm to global health. Modeling informed risk assessments and behaviors throughout the pandemic. The pandemic also provided an unprecedented level of seroprevalence data that identified and tested reliable modeling approaches [3,5–7]. Public health authorities and the broader scientific community should communicate this modeling success to the public. The public is otherwise less likely to trust reliable model-based risk assessments for future pandemics, especially when exposed to overly pessimistic assessments of model accuracy [25].

## Conclusion

Seroprevalence analyses shaped public understanding of pandemic risk and of the benefits of interventions such as vaccination. Mitigating sources of bias in seroprevalence studies better informs the public and pandemic preparedness plans. Authors should note the limitations of their serological studies to prevent misunderstanding by policymakers and the public. Commentators should also be cautious when interpreting early serological evidence from samples not representative of the general population or when extrapolating evidence from higher-income nations to lower-income nations. Epidemiological modeling and sampling of ongoing representative cohorts are an important corrective to early analyses based on nonprobability sampling. Preparation for future pathogen outbreaks should include establishment and maintenance representative cohorts, along with improved modeling and data bias adjustments.

## Data Availability

This work does not rely on any data or other resources beyond the publicly available sources cited.

## Abbreviations

CI: cumulative incidence
COVID-19: coronavirus disease 2019
IFR: infection fatality rate
IHR: infection hospitalization rate
SARS-CoV-2: severe acute respiratory syndrome coronavirus 2
WHO: World Health Organization
